# Supplementary material for: mTORC1 activation in lung mesenchyme drives sex- and age-dependent pulmonary structure and function decline
Source: Nat Commun. 2020 Nov 6;11:5640. doi: 10.1038/s41467-020-18979-4 (PMC7648630; doi:10.1038/s41467-020-18979-4)
Supplement: Supplementary file 1 — Supplementary Information [file 41467_2020_18979_MOESM1_ESM.pdf]

Supplementary Information

mTORC1 activation in lung mesenchyme drives sex- and age-dependent pulmonary structure and function decline

Kseniya Obraztsova, et al.

Alveolar Epithelial Cell clusters

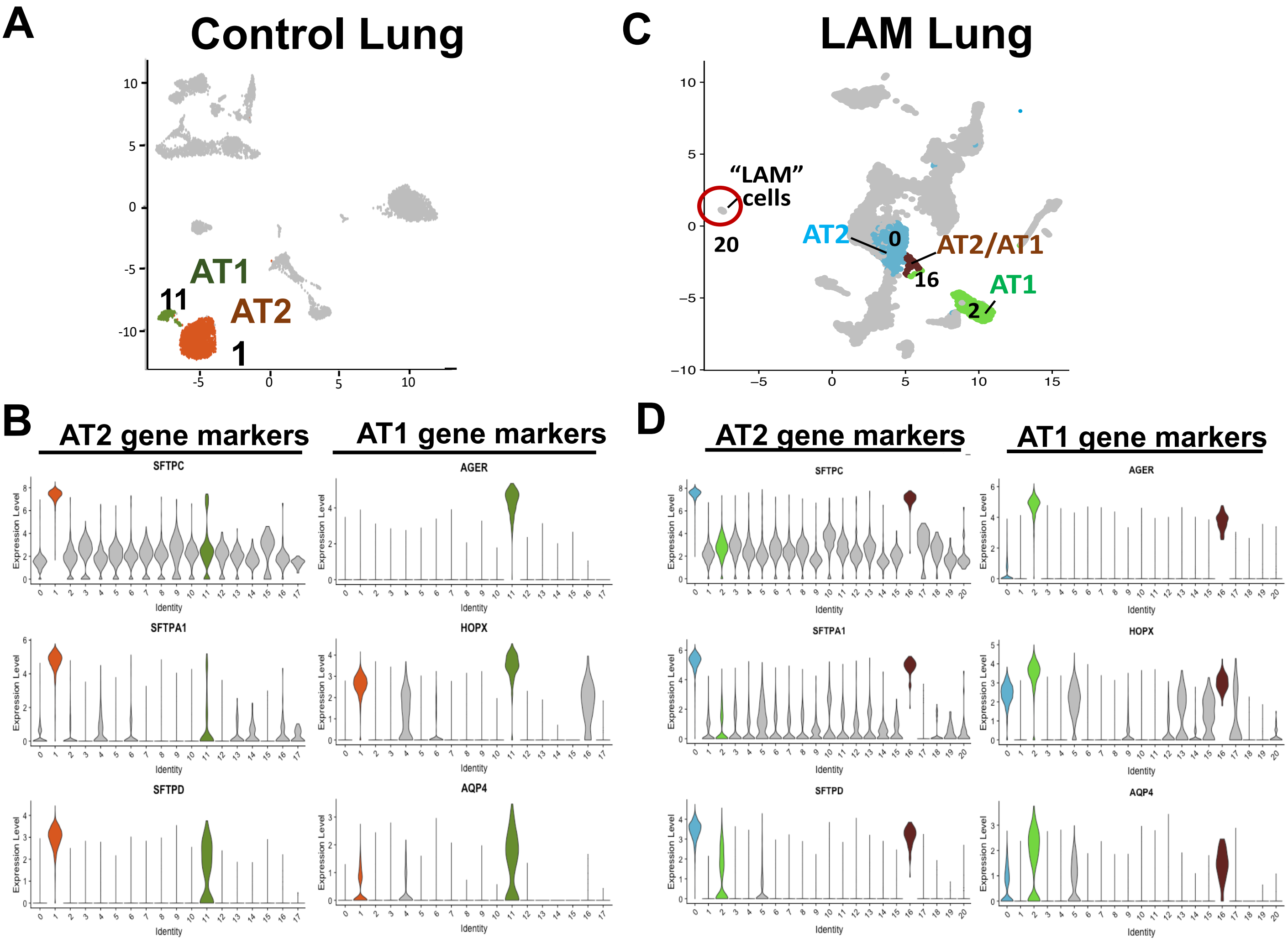

Supplementary Figure 1. Comparison of the alveolar epithelial cell subtypes in control and LAM lung.

A. UMAP dimensionality reduction plot of the control lung scRNA-seq cell clusters with highlighted only alveolar epithelial cell (AEC) clusters. Cluster 1 represents AT2 cells; cluster 11 – AT1 cells. **B.** Violin plots reflecting expression levels of the signature genes in the highlighted clusters of AT1 and AT2 cells. **C.** UMAP dimensionality reduction plot of the control lung scRNA-seq cell clusters with highlighted only alveolar epithelial cell (AEC) clusters. Cluster 0 represents AT2 cells; cluster 2 – AT1 cells, and cluster 16 represents transitional AT2/AT1 cell states. **D.** Violin plots reflecting expression levels of the signature genes in the highlighted clusters illustrate the simultaneous expression of AT1 and AT2 genes in cluster 16 (AT2/AT1 cells).

Mesenchymal cell clusters

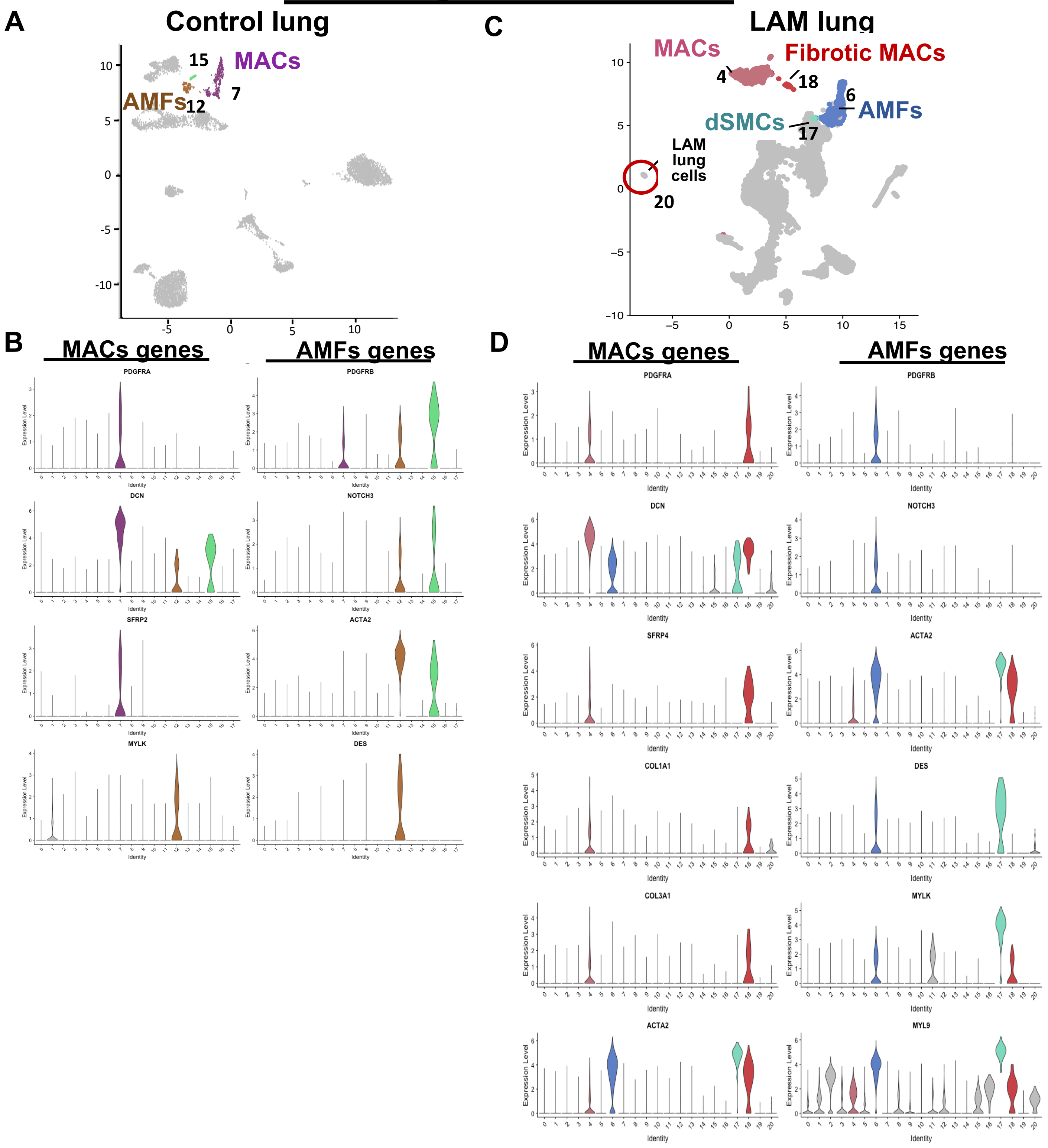

Supplementary Figure 2. Comparison of the mesenchymal cell subtypes in the control and LAM human lung. **A.** UMAP dimensionality reduction plot of the LAM lung scRNA-seq cell clusters (highlighted only mesenchymal clusters). Cluster 7 - mesenchymal alveolar cells (MACs); clusters 12 and 15– alveolar myofibroblasts (AMFs). **B.** Violin plots reflect the signature genes expression in the highlighted clusters. **C.** UMAP dimensionality reduction plot of the LAM lung scRNA-seq cell clusters (highlighted only mesenchymal clusters). Cluster 4 -mesenchymal alveolar cells (MACs); cluster 6 – alveolar myofibroblasts (AMFs), cluster 17 - fully differentiated smooth-muscle cells (dSMCs), and cluster 18 – fibrotic MACs. **D.** Violin plots illustrate specific co-expression of MACs signature genes with fibrotic gene markers *ACTA2, COL1A1, COL3A1* in the cluster 18, and the enhanced expression of SMCs genes *MYLK, MYL9, DES* combined with the loss of AMF markers *PDGFRB* and *NOTCH3* in the cluster 17.

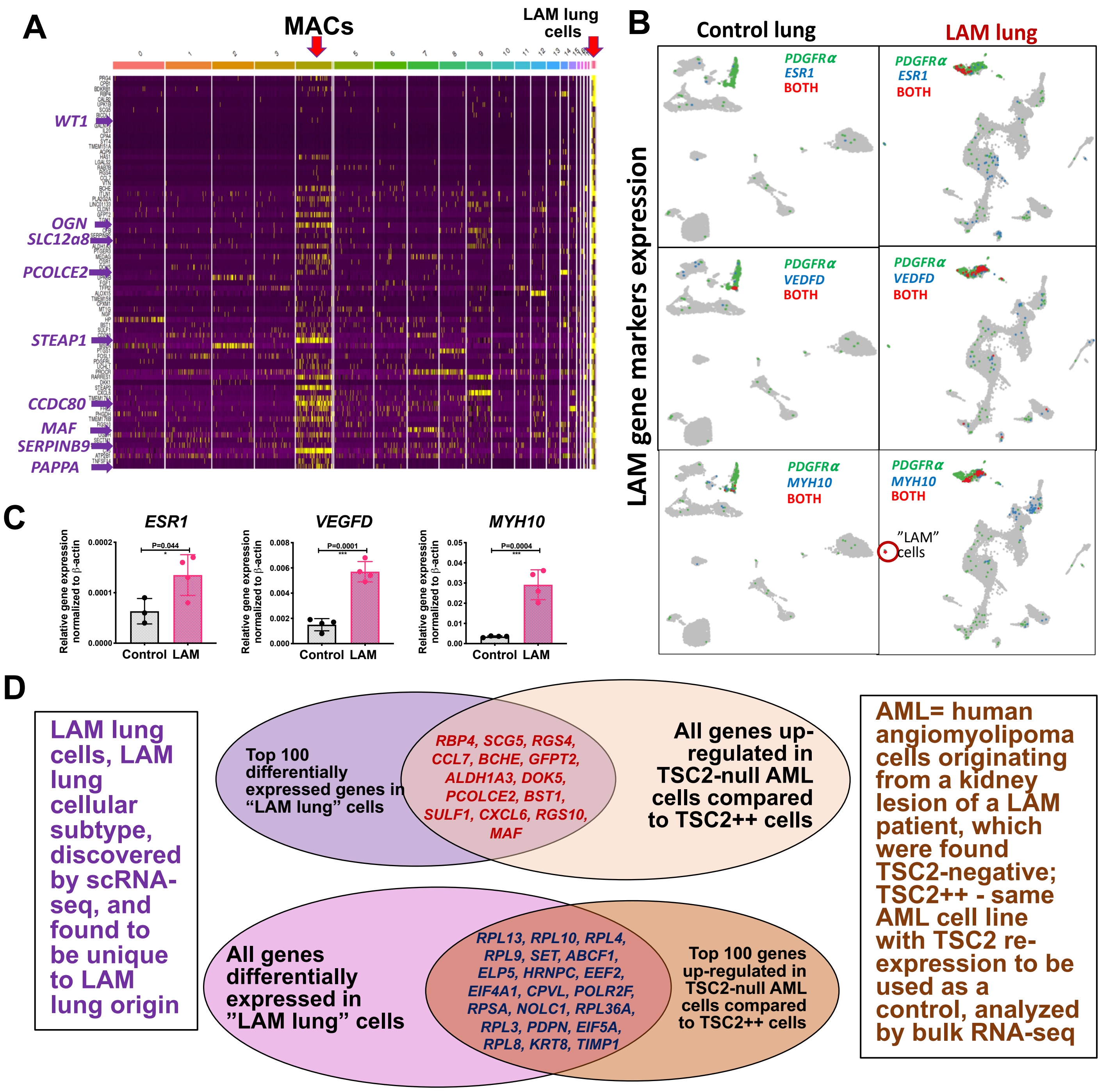

Supplementary Figure 3. LAM lung cell subset is the most transcriptionally close to MACs.
A. Top 100 differentially expressed genes in LAM lung cells subset compared to other scRNA-seq cell clusters in LAM lung. Highlighted genes are the potential transcriptional targets of the estrogen receptor (ERα). **B.** Increased gene expression of the LAM-associated genes *ESR1*, *VEGFD,* and *MYH10*. Side-by-side comparison of the dimensionality reduction plots (UMAPs) of scRNA-seq data from the control and LAM lung. Biplots represent the spatial expression of the two highlighted genes (one in blue and one in red) across the whole dataset. Overlap of the green and blue colors is shown in red, which indicates the simultaneous expression of the both marked gene within the cell cluster. The expression of *MYH10* gene partially correlates with LAM MACs and mostly correlates with LAM lung cells. **C.** Validation of the scRNA-seq data on selected genes using qPCR analysis of the mRNA isolated from the 3 control and 4 LAM lung samples. Graphs represent individual values, as well as means and SDs obtained using a parametric unpaired T-test. **D.** A comparison of the unique genes expressed in the newly discovered LAM lung cells and the upregulated geneset in the known human LAM cell line (TSC2-neg angiomyolipoma cells). Raw Data underlying figures in panels C and D is available in a Source Data File.

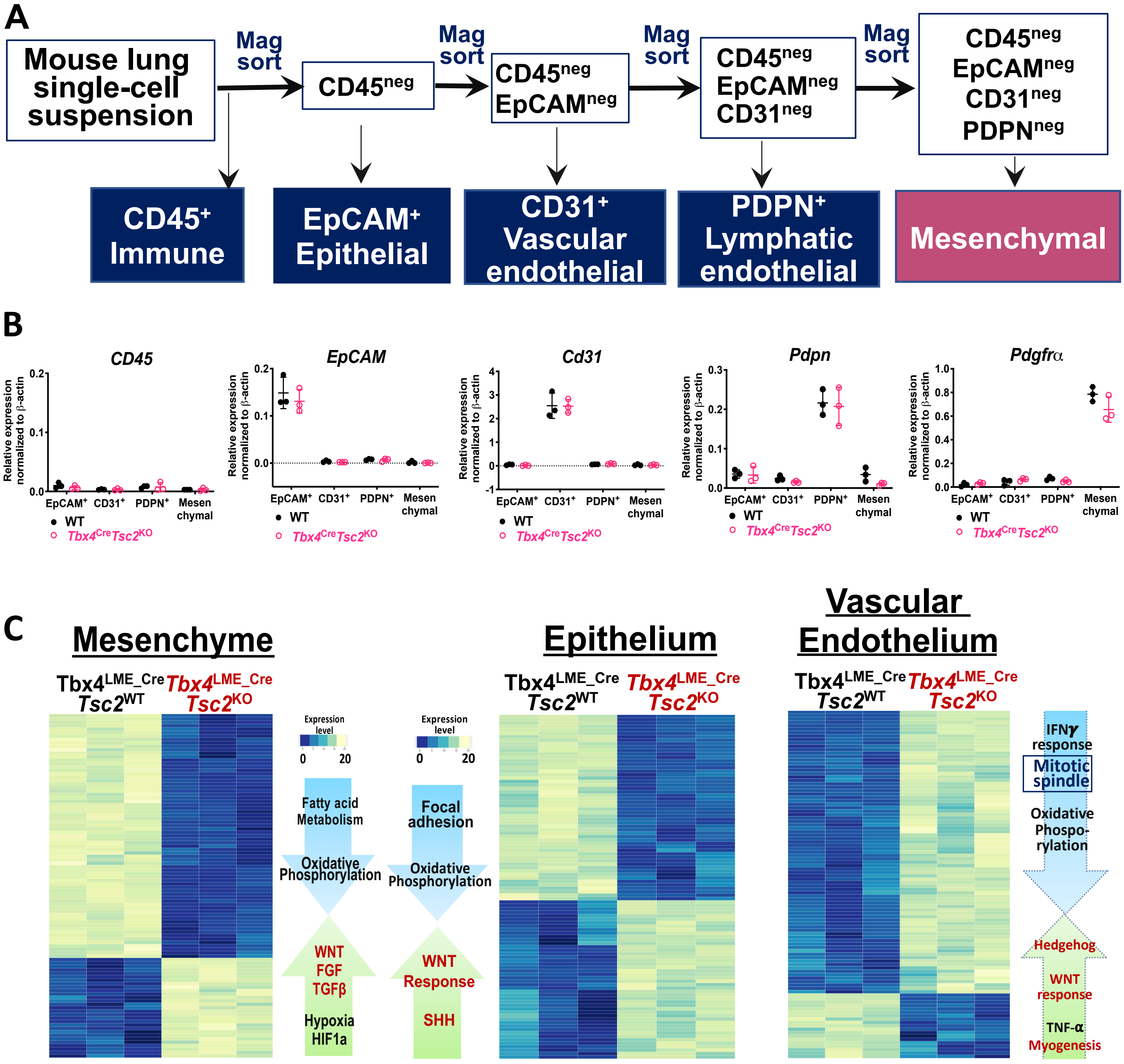

Supplementary Figure 4. Bulk RNA-seq of the sorted cell populations of the 8-week-old *Tbx4*$^{LME\_Cre}$*Tsc2*^{WT} and *Tbx4*$^{LME\_Cre}$*Tsc2*^{KO} mouse lungs. A. Experimental scheme of the consecutive magnetic sort of the mouse lung cell populations for the population RNA-seq analysis. **B.** Cell-type specific gene marker expression measured by qRT-PCR across the sorted cell populations. Presented as a quality control for the specificity of the cell-type selection during the sort procedure (N=3, error bars represent mean values with SD). **C.** Differential gene expression changes in the epithelial, vascular endothelial and mesenchymal cells isolated from the 8-week-old female *Tbx4*$^{LME\_Cre}$*Tsc2*^{WT} and *Tbx4*$^{LME\_Cre}$*Tsc2*^{KO} mouse lungs. Data obtained by the bulk RNA-seq on sorted cell populations (Pop-seq), (N=3). Heatmaps represent comparative expression levels for the individual replicates plotted side-by-side. Highlighted pathways (downregulated or upregulated, indicated by arrow) showed statistically significant enrichment during the GO term analysis of the top differentially expressed genes. Raw Data underlying figures in panel B is available in a Source Data File.

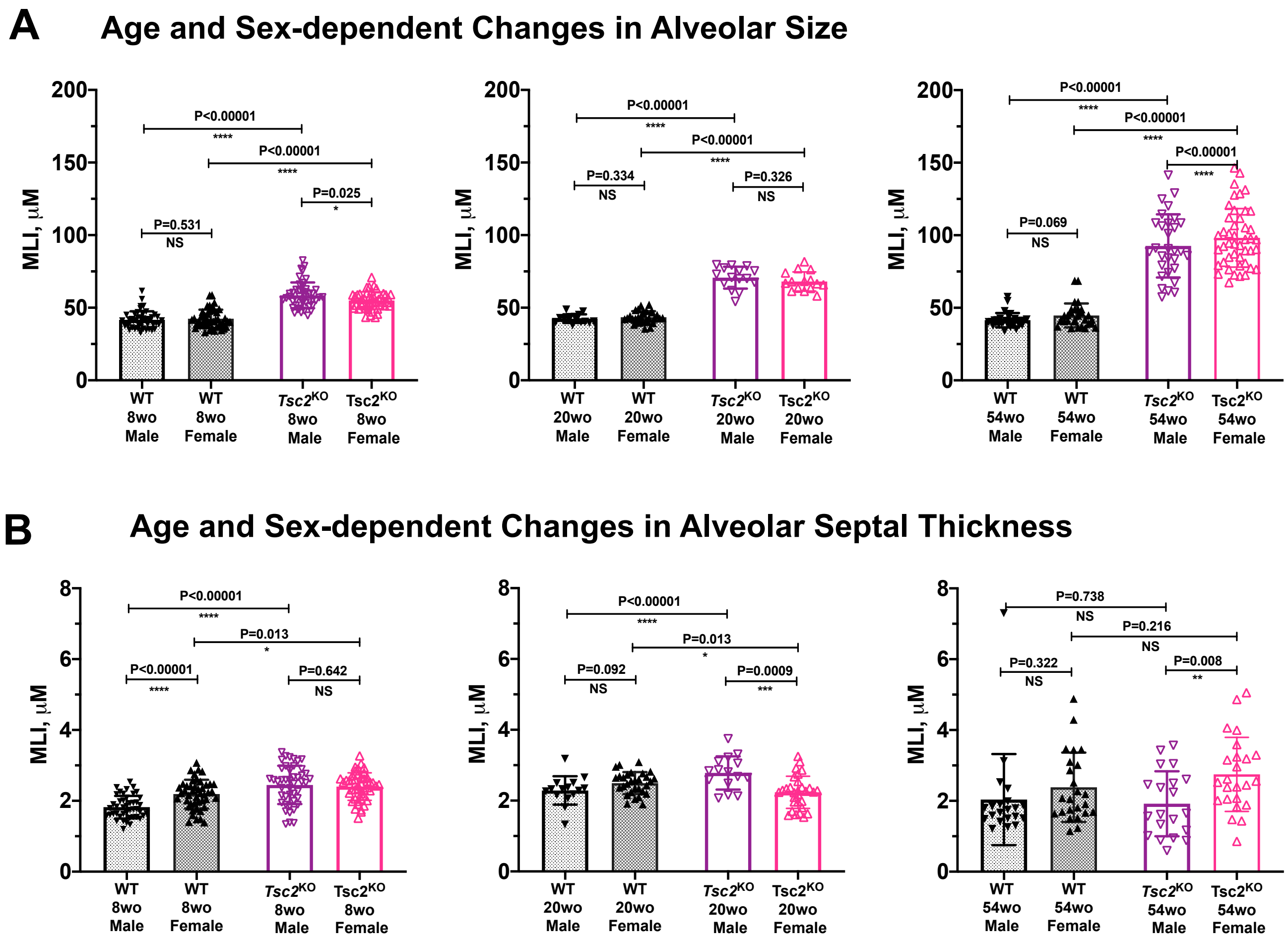

Supplementary Figure 5. Quantitative assessment of the age- and sex-dependent changes in the *Tbx4*$^{\text{LME\_Cre}}$*Tsc2*^{KO} mouse lung phenotype. A. Quantitation of the age- and sex-dependent changes of the mean alveolar size in *Tbx4*$^{\text{LME\_Cre}}$*Tsc2*^{KO} compared to *Tbx4*$^{\text{LME\_Cre}}$*Tsc2*^{WT} mouse lungs. Alveolar sizes are quantified using high throughput analysis of mean linear incepts (MLI), (N=4 per group, 15 images per lung sample). Graphs represent combined mean MLI measurements for all biological replicates within each experimental group. P-values, Means and SD errors were obtained using parametric two-tailed T-test. **B.** Alveolar Septal Thickness (AST) analysis in the 8-, 20-, and 54-week-old *Tbx4*$^{\text{LME\_Cre}}$*Tsc2*^{WT} and *Tbx4*$^{\text{LME\_Cre}}$*Tsc2*^{KO} mouse lungs. (N=4 for each group, 15 images per lung sample). Graphs represent combined repeated measurements for all biological replicates within each experimental group. P-values, Means and SD errors were obtained using multiple parametric two-tailed T-tests). Raw Data underlying figures in panels A and B is available in a Source Data File.

Supplementary Table 1. “LAM lung” cells and their gene signature.

The table illustrates the top unique and differentially expressed genes in “LAM lung” cells, cluster 20, with their correspondent biological role. The genes involved in urogenital development are highlighted in green, a number of genes regulating cellular proliferation are highlighted in orange, and genes associated with the ECM synthesis and remodeling are highlighted in blue.

Gene/Protein	Biological Role
**PRG4** (Proteoglycan 4)	cell membrane protein, plays a role as a growth factor acting on the primitive cells of both hematopoietic and endothelial cell lineages.
**CPB1** (carboxypeptidase B1)	peptide hormone metabolism, collagen chain trimerization
**BDKRB1** (bradykinin receptor B1)	inflammatory response
**CALB2** (calbindin 2)	calcium ion binding
**UPK1B** (uroplakin 1B)	bladder epithelium, membrane protein of urotehlial cells
**SCG5** (secretogranin V)	pituitary hormone secretion
**BICDL1** (BICD family like cargo adaptor 1)	component of secretory vesicle
**WT1** (WT1 transcription factor)	tumor supressor, urogenital system development
**GALNT9** (polypeptide N-acetylgalactosaminyltransferase 9)	mucin-type O-glycan biosythesis
**IL20** (interleukin 20)	Proinflammatory and angiogenic cytokine
**CPA4** (carboxypeptidase A4)	Metalloprotease that could be involved in the histone hyperacetylation pathway
**SYT4** (synaptotagmin 4)	plays a role in dendrite formation by melanocytes
**AQP9** (aquaporin 9)	Forms a water channel with a broad specificity
**HAS1** (hyaluronan synthase 1)	essential to hyaluronan synthesis a major component of most extracellular matrices that has a structural role in tissues architectures and regulates cell adhesion, migration and differentiation
**LGALS2** (galectin 2)	binds beta-galactoside
**RGS4** (regulator of G protein signaling 4)	inhibits signal transduction by increasing the GTPase activity of G protein alpha subunits thereby driving them into their inactive GDP-bound form
**CCL7** (C-C motif chemokine ligand 7)	Chemotactic factor that attracts monocytes and eosinophils, but not neutrophils
**RPRM** (reprimo, TP53 dependent G2 arrest mediator homolog)	involved in the regulation of p53-dependent G2 arrest of the cell cycle
**VTN** (vitronectin)	promotes cell adhesion and spreading, inhibits the membrane-damaging effect of the terminal cytolytic complement pathway
**ITLN1** (intelectin 1)	Increases AKT phosphorylation in the absence and presence of insulin
**CLDN15** (claudin 15)	integral membrane proteins and components of tight junction strands
**MGARP** (mitochondria localized glutamic acid rich protein)	Plays a role in the trafficking of mitochondria along microtubules.Also plays a role in steroidogenesis through maintenance of mitochondrial abundance and morphology
**EMB** (embigin)	involved in cell growth and development by mediating interactions between the cell and extracellular matrix.
**TGM1** (transglutaminase 1)	Catalyzes the cross-linking of proteins and the conjugation of polyamines to proteins. Involved in cell proliferation.
**SERPINB2** (serpin family B member 2)	Inhibits urokinase-type plasminogen activator(PAI-2). The monocyte derived PAI-2 is distinct from the endothelial cell-derived PAI-1
**SLC12A8** (solute carrier family 12 member 8)	Cation/chloride cotransporter that may play a role in the control of keratinocyte proliferation
**OSR1** (odd-skipped related transcription factor 1)	ranscription factor that plays a role in the regulation of embryonic heart and urogenital development
**DOK5** (docking protein 5)	provide a docking platform for the assembly of multimolecular signaling complexes, plays a positive role in activation of the MAP kinase pathway
**FGF1** (fibroblast growth factor 1)	regulation of cell survival, cell division, angiogenesis, cell differentiation and cell migration. Functions as potent mitogen
**TMEM158** (transmembrane protein 158 (gene/pseudogene))	Constitutive activation of the Ras pathway triggers an irreversible proliferation arrest reminiscent of replicative senescence. Transcription of this gene is upregulated in response to activation of the Ras pathway, but not under other conditions that induce senescence
**CDH6** (cadherin 6)	may play a role in kidney development as well as endometrium and placenta formation
**MPP6** (membrane palmitoylated protein 6)	Members of the peripheral membrane-associated guanylate kinase (MAGUK) family function in tumor suppression and receptor clustering by forming multiprotein complexes containing distinct sets of transmembrane, cytoskeletal, and cytoplasmic signaling proteins.
**ALDH1A2** (aldehyde dehydrogenase 1 family member A2)	catalyzes the synthesis of retinoic acid (RA) from retinaldehyde. Retinoic acid, the active derivative of vitamin A (retinol), is a hormonal signaling molecule that functions in developing and adult tissues.
**SULF1** (sulfatase 1)	This gene encodes an extracellular heparan sulfate endosulfatase. The encoded enzyme selectively removes 6-O-sulfate groups from heparan sulfate chains of heparan sulfate proteoglycans (HSPGs).
**FOSL1** (FOS like 1, AP-1 transcription factor subunit)	The Fos gene family consists of 4 members: FOS, FOSB, FOSL1, and FOSL2. These genes encode leucine zipper proteins that can dimerize with proteins of the JUN family, thereby forming the transcription factor complex AP-1. As such, the FOS proteins have been implicated as regulators of cell proliferation, differentiation, and transformation
**PDGFRL** (platelet derived growth factor receptor like)	encodes a protein with significant sequence similarity to the ligand binding domain of PDGFRB. Mutations in this gene, or deletion of a chromosomal segment containing this gene, are associated with sporadic hepatocellular carcinomas, colorectal cancers, and non-small cell lung cancers. This suggests this gene product may function as a tumor suppressor
**SMOC2** (SPARC related modular calcium binding 2)	matricellular protein which promotes matrix assembly and can stimulate endothelial cell proliferation and migration, as well as angiogenic activity. Associated with pulmonary function.

Supplementary Table 2. Antibodies used for protein detection.

ANTIBODY NAME	COMPANY	CAT#	ISOTYPE	REACTIVITY	PURPOSE	DILUTION
b-Actin	Cell Signaling	4967	rabbit	mouse	Western Blot	1:15000
pS6 (S235/236)	Cell Signaling	4856	rabbit	mouse	Western Blot	1:1000
TSC2	Cell Signaling	4308	rabbit	mouse	Western Blot	1:1000
mTOR	Cell Signaling	2983	rabbit	mouse	Western Blot	1:1000
pmTOR (Ser2448)	Cell Signaling	2976	rabbit	mouse	Western Blot	1:1000
S6	Cell Signaling	2217	rabbit	mouse	Western Blot	1:1000
4E-BP1	Cell Signaling	9425	rabbit	mouse	Western Blot	1:1000
p4E-BP1(Thr37/46)	Cell Signaling	2855	rabbit	mouse	Western Blot	1:1000
pMNK(Thr1897/202)	Cell Signaling	2115	rabbit	mouse	Western Blot	1:1000
MNK	Cell Signaling	2193	rabbit	mouse	Western Blot	1:1000
peIF4E(ser209)	Abcam	76256	rabbit	mouse	Western Blot	1:1000
pAKT(Ser473)	Cell Signaling	4060	rabbit	mouse	Western Blot	1:1000
**Secondary ab**						
**LiCOr800CW**	LiCor	926-32213	donkey	rabbit	Western Blot	1:15000
pS6 (S235/236)	Cell Signaling	mAb62016	mouse	Human Mouse Rat Monkey	IHC	1:200
PDGFRa	R&D systems	AF1062	goat	mouse	IHC	1:50
PDGFRb	Cell Signaling		rabbit	mouse	IHC	1:100
SPC	Santa Cruz		goat	mouse	IHC	1:50
HOPX	Santa Cruz	sc-398703	rat	mouse	IHC	1:200
aSMA	Sigma	A5228	mouse	mouse, human	IHC	1:400
PECAM1	Cell Signaling	3528	mouse	human	IHC	1:200
MYH10	Abcam	ab230823	rabbit	Mouse, Rat, Human	IHC	1:250
aSMA	Sigma	A2547	mouse	Human, Rat, Mouse	IHC	1:250
HT-I-56	Terrace Biotech	TB-29AHT1-56	mouse (IgG)	human	IHC	1:100
HT-II-280	Terrace Biotech	TB-27AHT2-280	mouse (IgM)	human	IHC	1:50
**Secondary ab**						
**anti-rabbit Alexa594**	Abcam	ab150080	goat	rabbit	IHC	1:250
**anti-rabbit Alexa488**	Abcam	ab150077	goat	rabbit	IHC	1:250
**anti-mouse Alexa594**	Abcam	ab150116	goat	mouse	IHC	1:250
**anti-mouse Alexa488**	Abcam	ab150113	goat	mouse	IHC	1:250
**anti-mouse Alexa594**	Jackson ImmunoResearch	AB_2338873	goat	mouse IgG (Fcγ fragment)	IHC	1:100
**anti-mouse Alexa488**	Abcam	ab150121	goat	mouse IgM (Mu chain)	IHC	1:100

Supplementary Table 3. Single-cell sequencing output results.

Sample	Repeat #	# of cells	Mean # of reads/cell	Median # of genes/cell
LAM	1	11,235	18,912	1,233
LAM	2	10,430	20,981	1,333
Control	1	7,343	28,054	1,298
Control	2	6, 382	31,649	1,242

Supplementary Table 4. DNA oligonucleotides used in the study for qPCR validation of RNA-seq data and for mouse genotyping.

**Primers used for human scRNA-seq validation**		
**Gene**	**Forward Primer sequence 5'-->3'**	**Reverse Primer sequence 5'-->3'**
ESR1	AGT GGC TTT GGT CCG TCT C	TCC TCA TCC TCT CCC ACA TC
VEGFD	TCG CTG TTC CCA TTC CAA GAA AC	CTG GTT CCT GGA GAT GAG AGT GGT C
MYH10	GCT GAT GGC AAC TCT CCG AAA C	CTT CCA GGA CAC CAT TAC AGC G
WNT2	AACAGAGCTGGCAGGAAG	AGAGATAATCGCCCGTTTTCC
FGF7	ATC AGG ACA TGT GCA GTT GGA	AAC ATT TCC CCT CCG TTG TGT
ACTNB	CAC CAA CTG GGA CGA CAT	ACA GCC TGG ATA GCA ACG
**Primers used for mouse RNA-seq validation**		
Actnb	CTG TCC CTG TAT GCC TCT G	ATG TCA CGC ACG ATT TCC
Cd45	GGG TTG TTC TGT GCC TTG TT	CTG GAC GGA CAC AGT TAG CA
Epcam	GAG TCC CTG TTC CAT TCT T	TCT CCT TTA TCT CAG CCT TC
CD31	GAA TGA CAC CCA AGC GTT TT	GGC TTC CAC ACT AGG CTC AG
Pdpn	GTG ACC CCA GGT ACA GGA GA	ATG GCT AAC AAG ACG CCA AC
Pdgfra	CAA ACC CTG AGA CCA CAA TG	TCC CCC AAC AGT AAC CCA AG
Tsc2	CAC TTT CGG AAG GCT GTC GTC TCA	AGA ACT CCC AAA CGA TCA CC
Hgf	AAA TGA GAA TGG TTC TTG GTG	CTG GCC TCT TCT ATG GCT
Wnt3a	CAC CAC CGT CAG CAA CAG CC	AGG AGC GTG TCA GAG AAG
Wnt4	GAG AAG TGT GGC TGT GAC CGG	ATG TTG TCC GAG CAT CCT GAC C
Wnt5a	CTC CTT CGC CCA GGT TGT TAT AG	TGT CTT CGC ACC TTC TCC AAT G
Fgf7	TTT GGA AAG AGC GAC GAC TT	GGC AGG ATC CGT GTC AGT AT
Fgf10	CAA CTC CGA TTT CCA CTG ATG T	GCT GTT CTC CTT CAC CAA GT
Fgf18	ACG TGG ATG CGG AAG TC	CCT GCA CTT GCC TGT GTT
Tgfb2	TCG ACA TGG ATC AGT TTA TGC G	CCC TGG TAC TGT TGT AGA TGG A
Col17a1	GAA AGG AGA CAA AGG TGA CCA	CGG CTT GAT GGC AAT ACT TC
Gli1	GGA AGT CCT ATT CAC GCC TTG A	CAA CCT TCT TGC TCA CAC ATG TAA G
Esr1	CGT GTG CAA TGA CTA TGC CTC T	TGG TGC ATT GGT TTG TAG CTG G
**Primers used for mouse genotyping**		
Tsc2	TCCGGCTTGAAGGAGAAGTT	ATTGTTGAGGCCGCATTCAC
**Generic Cre:**		
oIMR1084/5	GCGGTCTGGCAGTAAAAACTATC	GTGAAACAGCATTGCTGTCACTT
**Internal Positive Control**		
oIMR7338/9	CTAGGCCACAGAATTGAAAGATCT	GTAGGTGGAAATTCTAGCATCATC
