# Supplementary material for: mTORC1 activation in lung mesenchyme drives sex- and age-dependent pulmonary structure and function decline
Source: Nat Commun. 2020 Nov 6;11:5640. doi: 10.1038/s41467-020-18979-4 (PMC7648630; doi:10.1038/s41467-020-18979-4)
Supplement: Supplementary file 3 — Reporting Summary [file 41467_2020_18979_MOESM3_ESM.pdf]

nature research

Corresponding author(s): Vera Krymskaya, PhD

Last updated by author(s): Sep 15, 2020

Reporting Summary

Nature Research wishes to improve the reproducibility of the work that we publish. This form provides structure for consistency and transparency in reporting. For further information on Nature Research policies, see Authors & Referees and the Editorial Policy Checklist .

Statistics

For all statistical analyses, confirm that the following items are present in the figure legend, table legend, main text, or Methods section.

n/a	Confirmed	
☐	☒	The exact sample size (*n*) for each experimental group/condition, given as a discrete number and unit of measurement
☐	☒	A statement on whether measurements were taken from distinct samples or whether the same sample was measured repeatedly
☐	☒	The statistical test(s) used AND whether they are one- or two-sided<br>*Only common tests should be described solely by name; describe more complex techniques in the Methods section.*
☐	☒	A description of all covariates tested
☒	☐	A description of any assumptions or corrections, such as tests of normality and adjustment for multiple comparisons
☐	☒	A full description of the statistical parameters including central tendency (e.g. means) or other basic estimates (e.g. regression coefficient) AND variation (e.g. standard deviation) or associated estimates of uncertainty (e.g. confidence intervals)
☒	☐	For null hypothesis testing, the test statistic (e.g. *F*, *t*, *r*) with confidence intervals, effect sizes, degrees of freedom and *P* value noted<br>*Give P values as exact values whenever suitable.*
☒	☐	For Bayesian analysis, information on the choice of priors and Markov chain Monte Carlo settings
☒	☐	For hierarchical and complex designs, identification of the appropriate level for tests and full reporting of outcomes
☒	☐	Estimates of effect sizes (e.g. Cohen's *d*, Pearson's *r*), indicating how they were calculated

Our web collection on statistics for biologists contains articles on many of the points above.

Software and code

Policy information about availability of computer code

Data collection

For sequencing: CellRanger. For pulmonary function test: Scireq - flexiVent system. For imaging: Nikon Scope
RNA-seq: Fastqc v0.11.5; Picard 2.18; Star v2.5.2b; Rsubread;EdgeR; Limma (bioconductor v3.8)R 3.5
scRNA-seq: CellRanger v3.0.1;Seurat v3.0

Data analysis

Imaging and Analysis - Adobe Photoshop CS6, FIJI 2.0.0-rc-43/1.51f, Nikon Elements v4.60.00. Statistical Analysis - Graphpad Prism 8.4.3, MatLab R2020a.
The annotated codes for the high-throughput image analysis are available for public access in the GitHub Repository: https://github.com/aravind245/biovision

Data

Human scRNA-seq and bulk mouse lung cell population RNA-seq data that support the findings of this study have been deposited to the public functional genomics data repository Gene expression Omnibus (GEO). **Datasets have a "super-series" accession with the accession code GSE139819.** To review GEO accession GSE139819 please go to [https://www.ncbi.nlm.nih.gov/geo/query/acc.cgi?acc=GSE139819]
The source data underlying Figs 2f, 3b, 3c, 4b-d, 5a, 5c-d, 6b, 6d, 6f-h, 7a-c, 8a and 8b, and Supplementary Figs 3C, 4B, 5A and 5B are provided in a Source Data file. The Source Data file is supplemented with the manuscript.
The annotated codes for the high throughput image analysis of mouse lung MLIs and ASTs are available for public access in the GitHub repository: [https://github.com/aravind245/biovision].
The rest of the source data that support the findings of this study are available from the corresponding author upon reasonable request.

Field-specific reporting

Please select the one below that is the best fit for your research. If you are not sure, read the appropriate sections before making your selection.

☒ Life sciences ☐ Behavioural & social sciences ☐ Ecological, evolutionary & environmental sciences

For a reference copy of the document with all sections, see nature.com/documents/nr-reporting-summary-flat.pdf

Life sciences study design

All studies must disclose on these points even when the disclosure is negative.

Sample size	Sample sizes for animal studies were made as large as possible based on the complex genetics and long term nature of these experiments. As a baseline, N=3 was chosen empirically given the observed variation in response to mesenchymal *Tsc2* KO in a series of preliminary experiments. Many experiments in the manuscript have N=6 to ensure robustness. For all animal experiments, multiple animals were tested in 2-4 individual experiments to control for inter-cohort variation. Statistics were calculated across all biological and technical replicates. In experiments other than the animal studies, the sample size was predetermined based on preliminary assessment which provided the highest statistical power.
Data exclusions	No data were excluded.
Replication	There were no difficulties with reproduction of any of the reported findings. All experiments for this study are from multiple biological and technical replicates, with N and replication numbers reported in the legend of each Figure.
Randomization	For all animal experiments, experimental and control animals were chosen at random from age mated litters with the appropriate genotype. All experiments were performed with the appropriate age- and sex- matched wild-type control mice on a similar mixed background. All animals in these studies were maintained on a similar mixed C57Bl/6 x FVB genetic background. Both males and females were used in this study.<br><br>For human studies, control healthy samples from each individual were used as comparator for experimental manipulations on LAM lung samples. When possible, larger numbers of technical replicates were used given the range of human genetic variation. No randomization was possible for these samples.
Blinding	The genotype of all animals was known to the investigators at the outset of the studies as all animals in each experiment and treatment group were sex and age-matched with the appropriate genotype. Blinding to experimental condition was challenging as the *Tsc2* KO in the lung mesenchyme resulted in an obvious phenotypic changes in examined tissues.<br>Blinding to condition for quantitative counting was attempted but is not possible given the severity of the phenotype and the morphological differences in lung tissues.

Reporting for specific materials, systems and methods

We require information from authors about some types of materials, experimental systems and methods used in many studies. Here, indicate whether each material, system or method listed is relevant to your study. If you are not sure if a list item applies to your research, read the appropriate section before selecting a response.

Materials & experimental systems

n/a	Involved in the study	
☐	☒	Antibodies
☐	☒	Eukaryotic cell lines
☐	☐	Palaeontology
☐	☒	Animals and other organisms
☐	☒	Human research participants
☐	☐	Clinical data

Methods

n/a	Involved in the study	
☐	☐	ChIP-seq
☐	☐	Flow cytometry
☐	☐	MRI-based neuroimaging

Antibodies

Antibodies used	Extensive details of all antibodies (supplier name, catalog number, clone number)is available in the Methods section, as well as provided in the Supplementary table 4.
Validation	Multiple antibodies were obtained and tested at multiple concentrations in IHC. Staining was confirmed as specific by IHC in comparison to an isotype antibody or no primary antibody control.

Eukaryotic cell lines

Policy information about cell lines

Cell line source(s)	Primary mouse fibroblasts cell lines were obtained from either $Tbx4^{Cre}Tsc2^{WT}$ or $Tbx4^{Cre}Tsc2^{KO}$ mouse lung and used in mouse lung organoid experiments.

Authentication	Mouse primary cells were used at a low passage number and discarded after passage 6.
Mycoplasma contamination	All cells were tested negative for mycobacterial infection
Commonly misidentified lines (See ICLAC register)	No misidentified lines were used

Palaeontology

Specimen provenance	*Provide provenance information for specimens and describe permits that were obtained for the work (including the name of the issuing authority, the date of issue, and any identifying information).*
Specimen deposition	*Indicate where the specimens have been deposited to permit free access by other researchers.*
Dating methods	*If new dates are provided, describe how they were obtained (e.g. collection, storage, sample pretreatment and measurement), where they were obtained (i.e. lab name), the calibration program and the protocol for quality assurance OR state that no new dates are provided.*

☐ Tick this box to confirm that the raw and calibrated dates are available in the paper or in Supplementary Information.

Animals and other organisms

Policy information about studies involving animals; ARRIVE guidelines recommended for reporting animal research

Laboratory animals	In this study *Mus musculus* of BL6;*FVB-Tsc2*$^{flox/flox}$ *Tbx4*$^{LME\text{-}Cre}$ were used. The age used varied from 8-52 weeks of age. Both male and female mice were used. These mice were obtained by crossing *Tsc2*$^{loxP/loxP}$ mice from Stephen Hammes at University of Rochester, with *Tbx4*$^{LME\text{-}Cre}$ mice from Mark Krasnow at Stanford University. Original paper references: *Tsc2*$^{loxP/loxP}$ (PubMed ID:17245776), and *Tbx4*$^{LME\text{-}Cre}$ (PubMed ID 25395543). Mice were kept in CRB animal facility. Rooms ranged from 68-78F and 20-70% humidity. Lights are on 12hr cycle on 7a and off 7p year around.
Wild animals	The study did not involve wild animals.
Field-collected samples	The study did not involve samples collected from the field.
Ethics oversight	All animal studies were performed under guidance of the University of Pennsylvania Institutional Animal Care and Use Committee (IACUC).

Note that full information on the approval of the study protocol must also be provided in the manuscript.

Human research participants

Policy information about studies involving human research participants

Population characteristics	This study utilized tissue samples derived from de-identified non-utilized lungs donated for organ transplantation via an established protocol (PROPEL, approved by University of Pennsylvania Institutional Review Board) with informed consent in accordance with institutional procedures. All patient information was removed prior to use. This use does not meet the current NIH definition of human subject research, but all institutional procedures required for human subject research were followed throughout the reported experiments
Recruitment	**No participants were recruited**
Ethics oversight	**Lung tissue samples were obtained from human lung transplant donors signed informed consent, in accordance with the Declaration of Helsinki and approved by the I.Institutional Review Board at the University of Pennsylvania and NDRI**

Note that full information on the approval of the study protocol must also be provided in the manuscript.

Clinical data

Policy information about clinical studies

All manuscripts should comply with the ICMJEguidelines for publication of clinical research and a completedCONSORT checklist must be included with all submissions.

Clinical trial registration	*Provide the trial registration number from ClinicalTrials.gov or an equivalent agency.*
Study protocol	*Note where the full trial protocol can be accessed OR if not available, explain why.*
Data collection	*Describe the settings and locales of data collection, noting the time periods of recruitment and data collection.*
Outcomes	*Describe how you pre-defined primary and secondary outcome measures and how you assessed these measures.*

ChIP-seq

Data deposition

- [ ] Confirm that both raw and final processed data have been deposited in a public database such as GEO.
- [ ] Confirm that you have deposited or provided access to graph files (e.g. BED files) for the called peaks.

Data access links<br>*May remain private before publication.*	*For "Initial submission" or "Revised version" documents, provide reviewer access links. For your "Final submission" document, provide a link to the deposited data.*
Files in database submission	*Provide a list of all files available in the database submission.*
Genome browser session<br>(e.g. UCSC)	*Provide a link to an anonymized genome browser session for "Initial submission" and "Revised version" documents only, to enable peer review. Write "no longer applicable" for "Final submission" documents.*

Methodology

Replicates	*Describe the experimental replicates, specifying number, type and replicate agreement.*
Sequencing depth	*Describe the sequencing depth for each experiment, providing the total number of reads, uniquely mapped reads, length of reads and whether they were paired- or single-end.*
Antibodies	*Describe the antibodies used for the ChIP-seq experiments; as applicable, provide supplier name, catalog number, clone name, and lot number.*
Peak calling parameters	*Specify the command line program and parameters used for read mapping and peak calling, including the ChIP, control and index files used.*
Data quality	*Describe the methods used to ensure data quality in full detail, including how many peaks are at FDR 5% and above 5-fold enrichment.*
Software	*Describe the software used to collect and analyze the ChIP-seq data. For custom code that has been deposited into a community repository, provide accession details.*

Flow Cytometry

Plots

Confirm that:

- [ ] The axis labels state the marker and fluorochrome used (e.g. CD4-FITC).
- [ ] The axis scales are clearly visible. Include numbers along axes only for bottom left plot of group (a 'group' is an analysis of identical markers).
- [ ] All plots are contour plots with outliers or pseudocolor plots.
- [ ] A numerical value for number of cells or percentage (with statistics) is provided.

Methodology

Sample preparation	*Describe the sample preparation, detailing the biological source of the cells and any tissue processing steps used.*
Instrument	*Identify the instrument used for data collection, specifying make and model number.*
Software	*Describe the software used to collect and analyze the flow cytometry data. For custom code that has been deposited into a community repository, provide accession details.*
Cell population abundance	*Describe the abundance of the relevant cell populations within post-sort fractions, providing details on the purity of the samples and how it was determined.*
Gating strategy	*Describe the gating strategy used for all relevant experiments, specifying the preliminary FSC/SSC gates of the starting cell population, indicating where boundaries between "positive" and "negative" staining cell populations are defined.*

- [ ] Tick this box to confirm that a figure exemplifying the gating strategy is provided in the Supplementary Information.

Magnetic resonance imaging

Experimental design

Design type	*Indicate task or resting state; event-related or block design.*

Design specifications	*Specify the number of blocks, trials or experimental units per session and/or subject, and specify the length of each trial or block (if trials are blocked) and interval between trials.*
Behavioral performance measures	*State number and/or type of variables recorded (e.g. correct button press, response time) and what statistics were used to establish that the subjects were performing the task as expected (e.g. mean, range, and/or standard deviation across subjects).*

Acquisition

Imaging type(s)	*Specify: functional, structural, diffusion, perfusion.*
Field strength	*Specify in Tesla*
Sequence & imaging parameters	*Specify the pulse sequence type (gradient echo, spin echo, etc.), imaging type (EPI, spiral, etc.), field of view, matrix size, slice thickness, orientation and TE/TR/flip angle.*
Area of acquisition	*State whether a whole brain scan was used OR define the area of acquisition, describing how the region was determined.*

Diffusion MRI ☐ Used ☐ Not used

Preprocessing

Preprocessing software	*Provide detail on software version and revision number and on specific parameters (model/functions, brain extraction, segmentation, smoothing kernel size, etc.).*
Normalization	*If data were normalized/standardized, describe the approach(es): specify linear or non-linear and define image types used for transformation OR indicate that data were not normalized and explain rationale for lack of normalization.*
Normalization template	*Describe the template used for normalization/transformation, specifying subject space or group standardized space (e.g. original Talairach, MNI305, ICBM152) OR indicate that the data were not normalized.*
Noise and artifact removal	*Describe your procedure(s) for artifact and structured noise removal, specifying motion parameters, tissue signals and physiological signals (heart rate, respiration).*
Volume censoring	*Define your software and/or method and criteria for volume censoring, and state the extent of such censoring.*

Statistical modeling & inference

Model type and settings	*Specify type (mass univariate, multivariate, RSA, predictive, etc.) and describe essential details of the model at the first and second levels (e.g. fixed, random or mixed effects; drift or auto-correlation).*
Effect(s) tested	*Define precise effect in terms of the task or stimulus conditions instead of psychological concepts and indicate whether ANOVA or factorial designs were used.*

Specify type of analysis: ☐ Whole brain ☐ ROI-based ☐ Both

Statistic type for inference<br>(See Eklund et al. 2016)	*Specify voxel-wise or cluster-wise and report all relevant parameters for cluster-wise methods.*
Correction	*Describe the type of correction and how it is obtained for multiple comparisons (e.g. FWE, FDR, permutation or Monte Carlo).*

Models & analysis

n/a	Involved in the study
☐	☐ Functional and/or effective connectivity
☐	☐ Graph analysis
☐	☐ Multivariate modeling or predictive analysis

Functional and/or effective connectivity	*Report the measures of dependence used and the model details (e.g. Pearson correlation, partial correlation, mutual information).*
Graph analysis	*Report the dependent variable and connectivity measure, specifying weighted graph or binarized graph, subject- or group-level, and the global and/or node summaries used (e.g. clustering coefficient, efficiency, etc.).*
Multivariate modeling and predictive analysis	*Specify independent variables, features extraction and dimension reduction, model, training and evaluation metrics.*
